# Supplementary material for: Long-Term Impairment of Working Ability in Subjects under 60 Years of Age Hospitalised for COVID-19 at 2 Years of Follow-Up: A Cross-Sectional Study
Source: Viruses. 2024 Apr 26;16(5):688. doi: 10.3390/v16050688 (PMC11125725; doi:10.3390/v16050688)
Supplement: Supplementary file 1 [file viruses-16-00688-s001.zip › viruses-2953304-supplementary.pdf]

SUPPLEMENTAL MATERIAL

Table S1. Cumulative incidence of specific long COVID associated symptoms, stratified per duration of the follow-up period.

	**Follow-up ≤ 18 months (N=473)**	**Follow-up > 18 months (N=531)**	**Overall (N=1004)**
**Gender**			
F	204 (42.9%)	238 (44.8%)	442 (43.9%)
M	268 (56.7%)	291 (54.8%)	559 (55.7%)
Missing	2 (0.4%)	1 (0.2%)	3 (0.3%)
**Nationality**			
Italian	437 (92%)	491 (92.5%)	727 (72.4%)
Not Italian	36 (7.6%)	39 (7.3%)	75 (7.4%)
Missing	2 (0.4%)	1 (0.2%)	3 (0.3%)
**Age at admission**			
Mean (SD)	63.2 (18.0)	60.6 (17.2)	61.8 (17.6)
Median [Min, Max]	64.0 [16.5, 99.8]	61.5 [17.5, 98.7]	62.8 [16.5, 99.8]
Missing	1 (0.2%)	1 (0.2%)	2 (0.2%)
**Vaccinated**			
NO	160 (33.8%)	348 (65.5%)	508 (50.6%)
YES	313 (66.2%)	183 (34.5%)	496 (49.4%)
**Variant**			
Alpha	186 (39.3%)	132 (24.9%)	318 (31.7%)
Delta	116 (24.5%)	14 (2.6%)	130 (12.9%)
Omicron	141 (29.8%)	0 (0%)	141 (14.0%)
Wild-type	30 (6.3%)	385 (72.5%)	415 (41.3%)
**Admission to ICU**			
NO	295 (62.4%)	196 (36.9%)	491 (48.9%)

	Follow-up ≤ 18 months (N=473)	Follow-up > 18 months (N=531)	Overall (N=1004)
YES	30 (6.3%)	37 (7.0%)	67 (6.7%)
Missing	148 (31.3%)	298 (56.1%)	446 (44.4%)
NEUROLOGICAL SYMPTOMS			
**Headache**			
NO	272 (57.5%)	369 (69.5%)	641 (63.8%)
YES	201 (42.5%)	161 (30.3%)	362 (36.1%)
Missing	0 (0%)	1 (0.2%)	1 (0.1%)
**Taste disorder (ageusia or dysgeusia)**			
NO	433 (91.5%)	404 (76.1%)	837 (83.4%)
YES	40 (8.5%)	126 (23.7%)	166 (16.5%)
Missing	0 (0%)	1 (0.2%)	1 (0.1%)
**Smell disorder (anosmia)**			
NO	396 (83.7%)	368 (69.3%)	764 (76.1%)
YES	77 (16.3%)	162 (30.5%)	239 (23.8%)
Missing	0 (0%)	1 (0.2%)	1 (0.1%)
**Cognitive impairment**			
NO	278 (58.8%)	270 (50.8%)	548 (54.6%)
YES	115 (24.3%)	146 (27.5%)	261 (26.0%)
Missing	80 (16.9%)	115 (21.7%)	195 (19.4%)
**Memory deficits**			
NO	371 (78.4%)	338 (63.7%)	709 (70.6%)
YES	102 (21.6%)	192 (36.2%)	294 (29.3%)
Missing	0 (0%)	1 (0.2%)	1 (0.1%)
**Difficulty concentrating**			
NO	352 (74.4%)	389 (73.3%)	741 (73.8%)
YES	121 (25.6%)	139 (26.2%)	260 (25.9%)
Missing	0 (0%)	3 (0.6%)	3 (0.3%)
**Dizziness**			
NO	423 (89.4%)	478 (90.0%)	901 (89.7%)

	Follow-up ≤ 18 months (N=473)	Follow-up > 18 months (N=531)	Overall (N=1004)
YES	50 (10.6%)	51 (9.6%)	101 (10.1%)
Missing	0 (0%)	2 (0.4%)	2 (0.2%)
**Tremors**			
NO	444 (93.9%)	478 (90.0%)	922 (91.8%)
YES	29 (6.1%)	51 (9.6%)	80 (8.0%)
Missing	0 (0%)	2 (0.4%)	2 (0.2%)
**Visual impairment**			
NO	369 (78.0%)	413 (77.8%)	782 (77.9%)
YES	24 (5.1%)	3 (0.6%)	27 (2.7%)
Missing	80 (16.9%)	115 (21.7%)	195 (19.4%)
**PSICHIATRIC SYMPTOMS**			
**PTSD score**			
Mean (SD)	1.00 (0.973)	1.03 (1.31)	1.01 (1.15)
Median [Min, Max]	1.00 [0, 4.00]	0 [0, 5.00]	1.00 [0, 5.00]
Missing	8 (1.7%)	84 (15.8%)	92 (9.2%)
**Depression – HADS score**			
Mean (SD)	4.92 (3.99)	4.58 (4.44)	4.75 (4.21)
Median [Min, Max]	5.00 [0, 20.0]	5.00 [0, 21.0]	5.00 [0, 21.0]
Missing	10 (2.1%)	84 (15.8%)	94 (9.4%)
**Sleep disorder**			
NO	410 (86.7%)	473 (89.1%)	883 (87.9%)
YES	63 (13.3%)	58 (10.9%)	121 (12.1%)
**Anxiety – HADS score**			
Mean (SD)	5.77 (4.51)	5.26 (4.86)	5.50 (4.70)
Median [Min, Max]	4.00 [0, 18.0]	4.00 [0, 21.0]	4.00 [0, 21.0]
Missing	5 (1.1%)	1 (0.2%)	6 (0.6%)
**RESPIRATORY SYMPTOMS**			
**Cough**			
NO	381 (80.5%)	415 (78.2%)	796 (79.3%)

	Follow-up ≤ 18 months (N=473)	Follow-up > 18 months (N=531)	Overall (N=1004)
YES	92 (19.5%)	115 (21.7%)	207 (20.6%)
Missing	0 (0%)	1 (0.2%)	1 (0.1%)
**Dyspnea**			
NO	401 (84.8%)	434 (81.7%)	835 (83.2%)
YES	72 (15.2%)	96 (18.1%)	168 (16.7%)
Missing	0 (0%)	1 (0.2%)	1 (0.1%)
**Oxygen use**			
NO	471 (99.6%)	524 (98.7%)	995 (99.1%)
YES	2 (0.4%)	5 (0.9%)	7 (0.7%)
Missing	0 (0%)	2 (0.4%)	2 (0.2%)
**Voice change – VHI score**			
Mean (SD)	3.92 (3.19)	3.57 (3.20)	3.74 (3.20)
Median [Min, Max]	4.00 [0, 10.0]	3.00 [0, 10.0]	4.00 [0, 10.0]
Missing	0 (0%)	1 (0.2%)	1 (0.1%)
**Mobility impairment**			
NO	465 (98.3%)	525 (98.9%)	990 (98.6%)
YES	8 (1.7%)	5 (0.9%)	13 (1.3%)
Missing	0 (0%)	1 (0.2%)	1 (0.1%)
**Functional impairment**			
NO	471 (99.6%)	526 (99.1%)	997 (99.3%)
YES	2 (0.4%)	4 (0.8%)	6 (0.6%)
Missing	0 (0%)	1 (0.2%)	1 (0.1%)
**Weight loss**			
NO	444 (93.9%)	492 (92.7%)	936 (93.2%)
YES	29 (6.1%)	38 (7.2%)	67 (6.7%)
Missing	0 (0%)	1 (0.2%)	1 (0.1%)
**Myalgia**			
NO	430 (90.9%)	465 (87.6%)	895 (89.1%)
YES	43 (9.1%)	65 (12.2%)	108 (10.8%)
Missing	0 (0%)	1 (0.2%)	1 (0.1%)

	Follow-up ≤ 18 months (N=473)	Follow-up > 18 months (N=531)	Overall (N=1004)
**Pain**			
NO	389 (82.2%)	461 (86.8%)	850 (84.7%)
YES	84 (17.8%)	68 (12.8%)	152 (15.1%)
Missing	0 (0%)	2 (0.4%)	2 (0.2%)
**Fever**			
NO	463 (97.9%)	519 (97.7%)	982 (97.8%)
YES	10 (2.1%)	11 (2.1%)	21 (2.1%)
Missing	0 (0%)	1 (0.2%)	1 (0.1%)
**Fatigue**			
NO	324 (68.5%)	394 (74.2%)	718 (71.5%)
YES	149 (31.5%)	136 (25.6%)	285 (28.4%)
Missing	0 (0%)	1 (0.2%)	1 (0.1%)
**Arthralgia**			
NO	421 (89.0%)	459 (86.4%)	880 (87.6%)
YES	52 (11.0%)	71 (13.4%)	123 (12.3%)
Missing	0 (0%)	1 (0.2%)	1 (0.1%)
**Sore throat**			
NO	462 (97.7%)	515 (97.0%)	977 (97.3%)
YES	11 (2.3%)	14 (2.6%)	25 (2.5%)
Missing	0 (0%)	2 (0.4%)	2 (0.2%)
**Sweats**			
NO	471 (99.6%)	527 (99.2%)	998 (99.4%)
YES	2 (0.4%)	3 (0.6%)	5 (0.5%)
Missing	0 (0%)	1 (0.2%)	1 (0.1%)
**CARDIAC SYMPTHOMS**			
**Palpitation**			
NO	390 (82.5%)	447 (84.2%)	837 (83.4%)
YES	83 (17.5%)	82 (15.4%)	165 (16.4%)
Missing	0 (0%)	2 (0.4%)	2 (0.2%)
**Chest pain**			

	Follow-up ≤ 18 months (N=473)	Follow-up > 18 months (N=531)	Overall (N=1004)
NO	371 (78.4%)	415 (78.2%)	786 (78.3%)
YES	102 (21.6%)	114 (21.5%)	216 (21.5%)
Missing	0 (0%)	2 (0.4%)	2 (0.2%)
**Flushing**			
NO	466 (98.5%)	521 (98.1%)	987 (98.3%)
YES	7 (1.5%)	7 (1.3%)	14 (1.4%)
Missing	0 (0%)	3 (0.6%)	3 (0.3%)
**New onset hypertension**			
NO	471 (99.6%)	526 (99.1%)	997 (99.3%)
YES	2 (0.4%)	3 (0.6%)	5 (0.5%)
Missing	0 (0%)	2 (0.4%)	2 (0.2%)
**DIGESTIVE SYMPTOMS**			
**Abdominal pain**			
NO	426 (90.1%)	489 (92.1%)	915 (91.1%)
YES	47 (9.9%)	41 (7.7%)	88 (8.8%)
Missing	0 (0%)	1 (0.2%)	1 (0.1%)
**Diarrhea**			
NO	457 (96.6%)	499 (94.0%)	956 (95.2%)
YES	16 (3.4%)	31 (5.8%)	47 (4.7%)
Missing	0 (0%)	1 (0.2%)	1 (0.1%)
**Vomit**			
NO	471 (99.6%)	524 (98.7%)	995 (99.1%)
YES	2 (0.4%)	5 (0.9%)	7 (0.7%)
Missing	0 (0%)	2 (0.4%)	2 (0.2%)
**Loss of appetite**			
NO	446 (94.3%)	505 (95.1%)	951 (94.7%)
YES	27 (5.7%)	25 (4.7%)	52 (5.2%)
Missing	0 (0%)	1 (0.2%)	1 (0.1%)
**Rash**			
NO	465 (98.3%)	510 (96.0%)	975 (97.1%)

	**Follow-up ≤ 18 months (N=473)**	**Follow-up > 18 months (N=531)**	**Overall (N=1004)**
YES	8 (1.7%)	19 (3.6%)	27 (2.7%)
Missing	0 (0%)	2 (0.4%)	2 (0.2%)
**Hair loss**			
NO	446 (94.3%)	481 (90.6%)	927 (92.3%)
YES	27 (5.7%)	49 (9.2%)	76 (7.6%)
Missing	0 (0%)	1 (0.2%)	1 (0.1%)
